# Which teaching method is more effective in a communication course – role-playing versus simulated patients, taught by tutors or faculty staff? A randomized trial

**DOI:** 10.3205/zma001450

**Published:** 2021-03-15

**Authors:** Julia Herchenröther, Elena Tiedemann, Heiner Vogel, Anne Simmenroth

**Affiliations:** 1 Universitätsklinikum Würzburg, Institut für Allgemeinmedizin, Würzburg, Germany; 2 Universitätsklinikum und Universität Würzburg, Zentrum für Psychische Gesundheit, Arbeitsbereich Medizinische Psychologie und Psychotherapie, Würzburg, Germany

**Keywords:** medical history taking, communication skills, role-playing, simulated patients

## Abstract

**Aim: **Communicative skills can be taught and trained as part of medical training. In these courses, lecturers or tutors serve as instructors, and students perform interviews with simulated patients (SP) or role-play (RP) with fellow students. The present study was conducted to identify the combination of applied teaching methods that is most effective.

**Method: **n=144 third-year (5th semester) medical students attended a medical history-taking course that consisted of three separate sessions (90 minutes each). Students were randomized into three groups. Groups 1 and 2 received training with SP in sessions one and two, followed by RP training in session three; (teaching by lecturer = SP-Lecturer group vs. teaching by tutor = SP-Tutor group). Group 3 received two sessions of RP training and a SP-based training in session three (teaching by tutor = RP-Tutor group). After completing the course, students had to rate their learning success using a short questionnaire, pass an SP-OSCE station and were supposed to answer exam questions.

**Results:** The OSCE performance of both the SP-Lecturer group (n=28) and the RP-Tutor group (n=44) was significantly better than that of the SP-Tutor group (n=26; *p*=.018 and *p*=.041, respectively). All groups reported an increase in self-rated history-taking skills (*p*<.001). There was no difference between groups in the results achieved on the final exam.

**Conclusions: **Students receiving SP-based teaching benefit more from a lecturer-taught course, while students instructed by tutors benefit more from RP-based teaching. The significant learning progress highlights the great advantage of taking the course as part of medical studies. Further research should aim to determine at which time each teaching method improves learning progress most effectively.

## Zusammenfassung

**Zielsetzung:** Kommunikative Fertigkeiten können gelehrt und trainiert werden. In Anamnesekursen werden als Kursleitung Dozenten oder Tutoren mit Schauspielpatienten (SP) oder Rollenspiele (RP) mit Kommilitonen eingesetzt. Ziel dieser Studie ist es, die wirkungsvollste Kombination für die Lehre in einem Anamnesekurs zu evaluieren.

**Methodik: **Die Studierenden des Kurses „Praktische Klinische Untersuchungsmethoden" (*N*=144; 5. Semester) durchliefen einen dreiteiligen Anamnesekurs (3x 90 Minuten). Sie wurden in folgende Kleingruppen randomisiert: 2 Gruppen übten an zwei Terminen mit SP, an einem mit RP und wurden entweder von einem Dozent (SP-Dozent-Gruppe) oder von einem Tutor (SP-Tutor-Gruppe) unterrichtet; eine dritte Gruppe lernte mit Tutoren und vorwiegend RP (RP-Tutor-Gruppe). Am Ende des Kurses wurden der selbsteingeschätzte Lernerfolg mittels Fragebogen erhoben, Klausurfragen ausgewertet und eine Anamnese-OSCE-Station absolviert.

**Ergebnisse:** Die OSCE-Leistungen der SP-Dozent-Gruppe (N=28) waren signifikant besser als in der SP-Tutor-Gruppe (*N*=26; *p*=.018). Auch die RP-Tutor-Gruppe erreichte signifikant höhere OSCE-Werte als die SP-Tutor-Gruppe (*N*=44; *p*=.041). Alle Gruppen schätzten ihre Anamnese-Fertigkeiten retrospektiv als verbessert ein (*p*<.001), wobei sich keine Gruppenunterschiede im Gesamtlernzuwachs ergaben. Bei den Klausurergebnissen gab es keine Gruppenunterschiede.

**Schlussfolgerung: **Studierende, die mit SP trainieren, profitieren eher von der Kursleitung durch Dozenten, die Tutoren-Gruppe eher vom Einsatz von RP. Der gesteigerte Lernzuwachs in allen Gruppen unterstreicht den Stellenwert des Anamnesekurses. Weitere Studien könnten sich auf die Bestimmung des effektivsten Zeitpunkts für den Einsatz der unterschiedlichen Modalitäten konzentrieren.

## 1. Background

Communicative skills can be taught and trained [1]. Communication training has been integrated as structured courses in many undergraduate medical curricula [2]. The taking of a medical history and patient-centered communication have many positive effects: They increase patient satisfaction [3], raise the level of compliance [4] and encourage a health-related quality of life [5]. Both university lecturers and peer tutors have proven to be effective in teaching.

According to Topping et al., “tutors are people from similar social groupings, who are not professional teachers, helping each other to learn and by so doing, learning themselves” [6]. Tutors and their student peers share social and cognitive factors in common. Most of all, students value the relaxed learning environment that is created by tutor-based teaching [7]. However, in the literature no uniform consensus dominates regarding the superiority of lecturers or tutors. While tutors generally represent an effective choice for economic reasons [7], [8], in some studies they have in part been shown to be inferior when directly compared to lecturers in terms of self-assessed learning success and quicker attainment of learning objectives [9], [10]. In a meta-analysis, Rees et al. were unable to determine any differences in regard to knowledge and skills, whereby the authors noted that only a few studies on this topic exist as of yet and they yield some contradictory results [11]. In a systematic review, Yu et al. found that in 10 of 12 studies there was no significant difference in effectiveness between tutors and lecturers [7]. The two dissenting studies focused on topics apart from teaching communication skills, with one finding advantages to the use of lecturers [9] and the other to the use of tutors [12]. Although Nestel and Kidd were unable to objectively measure any differences in a communication course in which students took case histories with simulated patients (SP), they did find that significantly more students from the lecturer-led group reported having fully achieved multiple learning objectives and that SPs were more satisfied with the performance of the students in the peer-led group [10].

Schmidt et al. investigated a course with problem-oriented learning (POL): The groups that received instruction from lecturers scored higher on a written test. In addition, when surveyed a higher percentage of the students in the lecturer-taught group agreed with the statements that instruction given to groups by lecturers contributes relevant information and applies medical expertise in the teaching of knowledge [13]. In other places it was pointed out by students that tutors often had difficulty when teaching because of their lack of experience [14].

A variety of teaching methods are used to teach communication skills: real patients, simulated patients and role-playing (RP) with peers. SP are laypersons or actors who have been professionally trained to simulate symptoms, personality traits and to give a specific medical history [15]. Communication training with SP is effective [16], just as role-playing is with fellow students [17], [18]. Communication skills can be validly assessed in practical tests (e.g. taking a medical history during an OSCE) or on written tests (knowledge about the principles of doctor-patient communication).

There is a lack of consensus in the literature when directly comparing the effectiveness of teaching communication skills with SP or RP. On the one hand, no significant difference has been described between the two modalities [19], [20]; on the other, both the superiority of SP [21] and of RP have been shown [22]. Thus, not only SP, but also RP appears to be effective, although a mixing of both methods has hardly been studied at all. In the study undertaken by Bosse et al., medical students attended three training sessions with SP or RP and received structured feedback. Theoretical input was given in advance in each seminar. Both groups performed significantly better on an OSCE than a control group, with the RP group performing best. This seems to suggest that it is precisely a combination with increased use of RP in three sessions that leads to a better OSCE performance.

However, student performance in these studies was investigated using only RP or SP. Given this background, a study was initiated at the Würzburg University Hospital (UKW) as part of the medical history-taking course (PKU/Practical clinical examination techniques) that aimed to establish if lecturers or tutors led to better results in student performance. The study also aimed to investigate if the use of simulated patients or role-playing with fellow students led to better communication skills in the students.

The curriculum at UKW places value on teaching communication skills early. Already during the second semester, initial knowledge about the doctor-patient relationship is imparted in seminars and courses on medical psychology and sociology, and students become familiar with their role as physician not only using RP, but also in class sessions with SP. During the third and fourth semesters, an elective subject focused on psychiatric anamnesis with real patients can be taken. Later, required courses are taken that include a brief intervention for risky alcohol consumption/smoking, how to bear bad news, and how to communicate with family members of palliative patients about changes in the aim of care, as well as a seminar covering patient perspectives on breast cancer during which a patient from a self-help group is invited to speak to the students.

The communication course that is the subject of this study was held during the fifth semester. Each student attended 3x90 minutes in lectures and small-group settings. Previously the course had been taught by both tutors and lecturers, and both SP and RP were used. The present study aimed to systematically analyze the different methods on the basis of the following hypotheses:

1. Students who were taught by lecturers using SP (SP-Lecturer group) will achieve better scores on the OSCE and will rate their learning success higher in the self-assessment than students in the SP-Tutor group.
2. Secondary hypothesis: The RP-Tutor group will achieve higher scores on the OSCE than the SP-Tutor group. Also, the students in the RP-Tutor group will rate their learning success higher in the self-assessment than students in the SP-Tutor group.

Investigated in addition to this was whether student satisfaction with teaching by lecturers differs from the satisfaction of students taught by tutors, and whether differences exist regarding theoretical knowledge of history taking and communication as demonstrated in the responses to exam questions at the end of the semester.

## 2. Methods

### 2.1. Instruments for data collection

#### 2.1.1. Questionnaire

**Figure 1:**
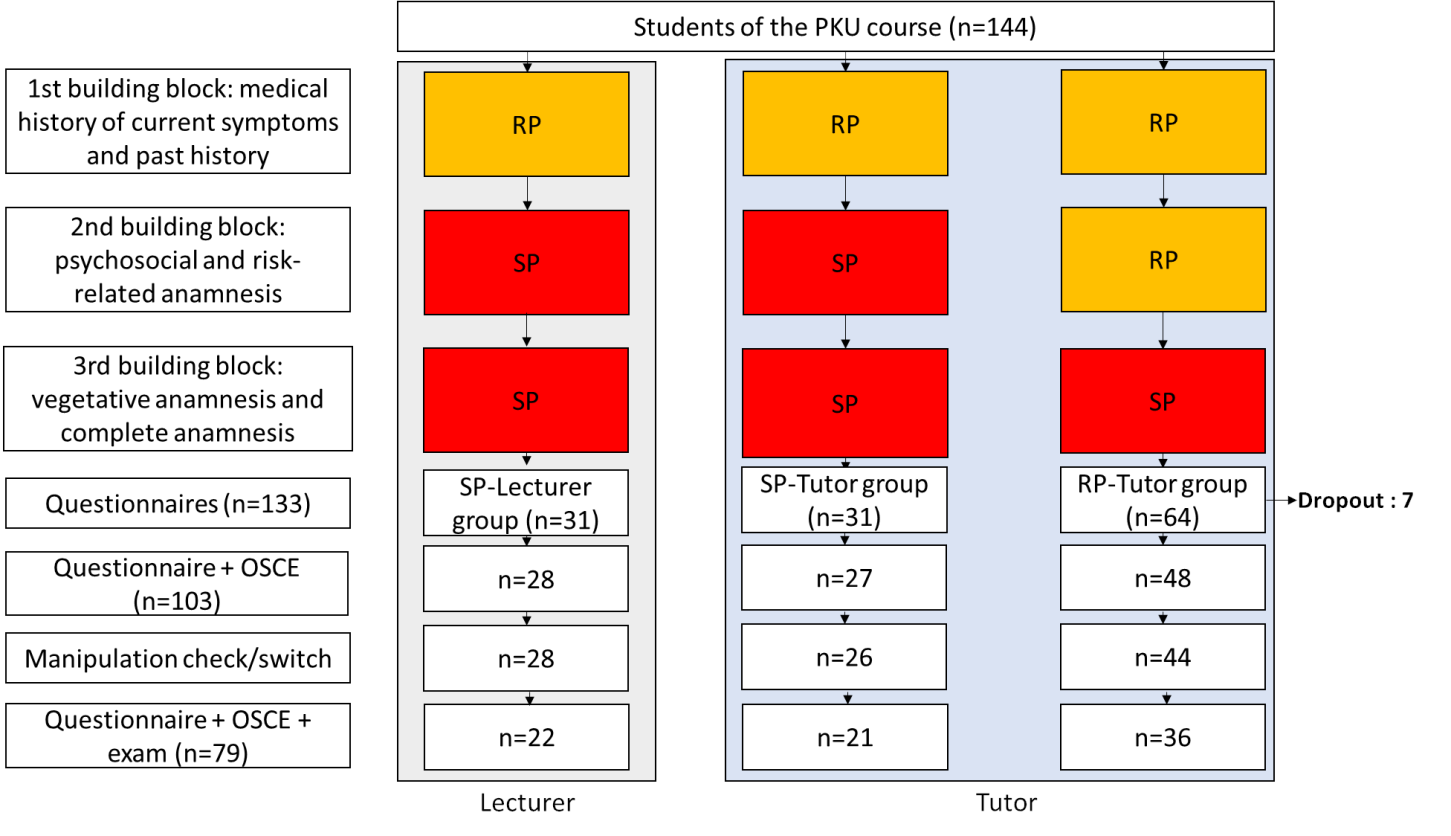
Flowchart of the study (Course of study. (SP = simulated patient, RP = role-playing with fellow students From left to right: SP-Lecturer, SP-Tutor, RP-Tutor group))

At the last meeting of each medical history-taking course the students were asked to fill out a voluntary survey (Questionnaire in attachment 1 ). Along with sociodemographic information such as sex, age, semester level, prior education (training in an occupation connected to medicine, clinical training), students were asked which small group they had been assigned to (SP-Lecturer, SP-Tutor or one of the RP-Tutor groups), if a lecturer or tutor had taught their group, if the training during the second session had been done using role-play or simulated patients, and if they had switched to another small group (see figure 1 (Fig. 1)).

**Figure 2:**
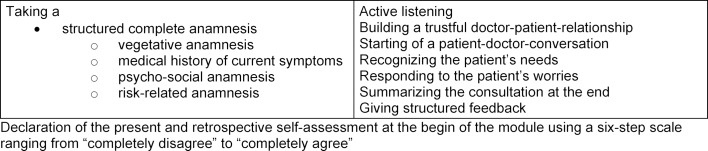
Enquired module’s communicative skills in the questionnaire

Questions pertaining to learning objectives (topic of anamnesis, see figure 2 )(Fig. 2), to differentiated evaluations of the course and to individual learning success (see figure 2 (Fig. 2) and Questionnaire in attachment 1 ) were asked using a six-step scale ranging from “completely disagree” to “completely agree.” A six-digit code generated by the students served to classify the questionnaires. The completed questionnaires were locked up at the Institute for General Practice, scanned and then saved on a password-protected computer at the Institute and were only accessible to the study authors.

##### 2.1.2. Test questions

An electronic questionnaire directly following a regularly scheduled electronic exam contained, in addition to asking for a student’s individual code and the assigned small group, three exam questions to be answered voluntarily. These questions referred to the script for the history-taking course:

1. The “seven dimensions of a symptom” when taking the history of symptoms (supplementation of two missing dimensions, open-ended questions);
2. The components of vegetative anamnesis (dropdown question with two response options);
3. A suitable question to ask when taking a medical history (multiple-choice, “1 of 5”).

##### 2.1.3. OSCE checklist

The required Würzburg curriculum calls for completion of a six-station OSCE after the fifth semester in which one station assesses history-taking skills. During the semester in which this study was conducted, this station focused on risk-related history taking (nicotine use, alcohol consumption). Eight minutes were allotted for this station, including the time needed to read the assigned task. All of the students encountered the same setting (same SP role, different SP). In addition to the general items from the Calgary-Cambridge Observation Guide [23], which are part of all OSCEs, the examiners also evaluated additional items on risk-related medical history taking. Each of these 23 dichotomies (yes/no) or trichotomies (yes/partially/no) were rated with a maximum of one point, added together to reach a total number of points, and then converted into a percent score (0-100%). Fifteen relevant items were recorded for the risk-related history-taking part of the station (smoking, alcohol, occupation, etc.). The examiners and SPs were trained in advance by the author who is listed last.

#### 2.2. Course sequence

The three building blocks of the history-taking course were taught in three separate course sessions (see figure 1 (Fig. 1)). The study was presented in detail during an introductory session for the semester as a way to inform the students and tutors about the study and how it would be conducted.

The semester cohort of 144 students was divided into seven groups of 24 students each, which were then randomly divided again into four small groups of six students each. The assignment to the small sub-groups was done randomly by using the colors of the students’ processing slips. All three course sessions were attended in the assigned groups so that the study manipulations could be traced. The course was organized so that the four small groups held the same number of history-taking interviews with SP or fellow students (as role-play) and were led either by a lecturer (physician or psychologist) or tutor (medical student with six or more semester of study). For logistical reasons, two of the four small groups received the same manipulation (instruction by a tutor, RP in two of three sessions) (see figure 1 (Fig. 1)). The small groups were usually taught by different lecturers or tutors in the three sessions, but the group assignment to lecturer-taught or tutor-taught, as well as the composition of the small groups, remained unchanged.

Both the SPs and the students who served as patients in the role-playing received instructions for the performances in advance. Two to three run-throughs were held in each RP session; in the SP sessions the students had contact with two SPs.

The Ethics Commission at the University of Würzburg approved the study (20190408 02).

#### 2.3. Data processing and analysis

The following statistical tests were applied: for the group differences on the OSCE and exam: Mann-Whitney U-test; for the analysis of the group differences in learning success: Welch’s t-test; for pre-post comparison: dependent sample t-test; for analysis of relationships: Spearman’s rank correlation coefficient. All tests of the hypothesis were one-sided, with a probability of error of *p*<.05 without adjusting for multiple testing. Effect sizes are given as Cohen’s d. Group differences regarding demographic aspects were analyzed, depending on applicability, with the chi-squared test, t-test or a one-way analysis of variance. All of the analyses were carried out using SPSS, version 25.0.

To measure the subjective learning success, a formula was used that corrects the pre-test bias [24]. All of the questionnaire items pertaining to learning success were aggregated into an overall gain in learning. Excluded from the analysis were extreme outliers concerning overall learning gain or incomplete responses to the questions on learning success.

Complete datasets from the questionnaire and OSCE served as the basis for analyzing the hypotheses and the demography; complete datasets from the questionnaire, OSCE and exam were drawn upon for analysis using the exam data. The manipulation check used to classify students according to their assigned groups enabled the exclusion of participants who indicated assignment to a small group that did not match the information they gave about being taught by lecturers or tutors.

## 3. Results

### 3.1. Preparatory data analysis

A total of 144 students attended the course, 133 filled out the questionnaire (response rate: 92.4%), 131 consented to participate in the study. One small group of seven students that was meant to be a RP-Tutor group had to be excluded from the analysis because it was – mistakenly – taught by a tutor only during the first two sessions.

Codes from the questionnaires and OSCE were classifiable in 103 cases. Four people had to be excluded because they switched to another group and another person was excluded for giving inaccurate information as detected by the manipulation check so that, in the end, 98 complete datasets from the questionnaire and OSCE were available. In the analysis of learning success another four people were excluded for not answering the related items completely or for being extreme outliers. Complete datasets from the questionnaire-OSCE-exam were available for 79 individuals, corresponding to 55% of the original sample. For six people the color listed for the small group did not match the color for the exam and questionnaire. An exclusion of these people did not result in any relevant changes in the exam results, which is why they were allowed to remain in the sample.

#### 3.2. Sample description

The majority of the sample were fifth-semester medical students (90%), mean age 23.4 years and predominantly female (63%). Prior to medical study, 26.8% of the students had completed training in an occupation connected with medicine, but only 7.2% had already undergone medical training in the clinical setting.

The two RP-Tutor groups (*N*=25; *N*=19) did not differ from each other in regard to sex (*p*=.632), age (*p*=.951), or previous training and education (*p*=.547), and were therefore combined into one group for the following analyses.

An overview of the demographic characteristics of the subgroups is presented in table 1 (Tab. 1). The SP-Lecturer, SP-Tutor, and RP-Tutor groups did not differ in regard to previous education or age, but they did differ in terms of distribution of the sexes, Χ^2^(2)=7.094, *p*=.029. If the comparative groups are viewed more closely, there is a difference between SP-Lecturer and SP-Tutor in regard to distribution of the sexes, Χ^2^(1)=6.865, *p*=.009, but not for the comparison between SP-Tutor and RP-Tutor, Χ^2^(1)=0.793, *p*=.373. Overall, there were no differences in OSCE performance between men and women, *p*=.485.

#### 3.3. Difference in OSCE performance for SP-based groups

**Figure 3:**
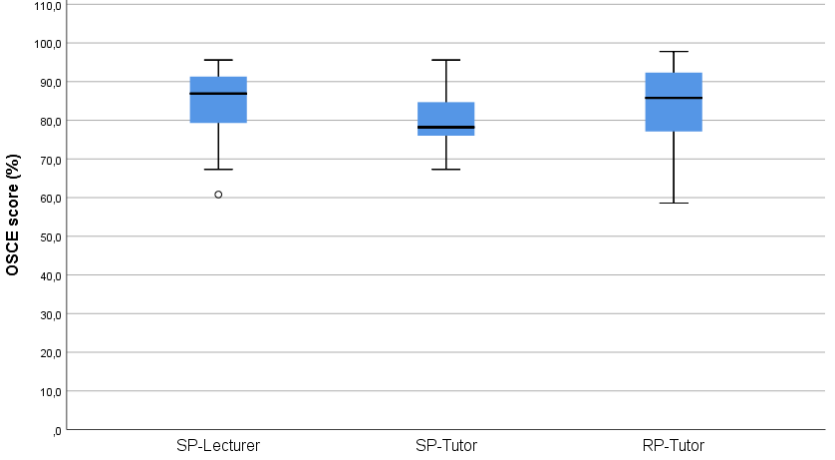
OSCE results of the three groups

OSCE performance of students in the SP-Lecturer group (Mdn=86.9%) was significantly better than that of the students in the SP-Tutor group (*Mdn*=78.2%), *U*=244.0, *z*=-2.09, *p*=.018, *d*=0.59 (see figure 3 (Fig. 3)).

If the individual OSCE categories are considered more closely, then only the difference in the part of the OSCE addressing “psychosocial and risk-related anamnesis” was significant, *U*=203.0,* z*=-2.80, *p*=.005, *d*=0.82. Out of a possible 14 points, the median point value for the SP-Lecturer group was 12.5 and for the comparative group 11. Particularly clear was the difference in the item “offering the option to quit smoking:” 89.3% of the SP-Lecturer group offered assistance to quit smoking, but only half of the students from the SP-Tutor group did.

In regard to overall learning success there was no significant difference between the SP-Lecturer group (*M*=20.6%, *n*=26) and the SP-Tutor group (*M*=24.2%, n=25), *t*(47)=-1.23, *p*=.22. The SP-Lecturer group retrospectively rated their skills in history taking as being higher after the course (*M*=84.1%) than before (*M*=67.8%), *t*(25)=-9.5, *p*<.001. The same applies to the SP-Tutor group (*M*=84.0% vs. *M*=62.2%), t(24)=-7.9, *p*<.001.

#### 3.4. Differences within the tutor-led groups

The RP-Tutor group (*Mdn*=85.8%) achieved higher OSCE scores than the SP-Tutor group (*Mdn*=78.2%), *U*=429.5, *z*=-1.74, *p*=.041, *d*= 0.42 (see figure 3 (Fig. 3)).

The scoring of the items on risk-related and psychosocial anamnesis showed a somewhat more marked difference between these two groups, *U*=363.5, *z*=-2.55, *p*=.011, *d*=0.64. No differences were seen in the other categories.

The overall learning success did not differ significantly between the SP-Tutor group (*M*=24.2%, *N*=25) and RP-Tutor group (*M*=21.3%,* N*=43), *t*(48)=1.03, *p*=.31. Just like the students in the SP-Tutor group, those in the RP-Tutor group rated their history-taking skills as being higher after the course than before the course (*M*=66.9% vs. *M*=85.0%), *t*(42)=-10.4, *p*<.001.

#### 3.5. Gain in knowledge and skills

All of the self-assessed skills in regard to medical history taking showed learning progress, *p*<.001. The smallest increase was seen in the learning success in “giving feedback” and “active listening;” the largest increase was in “taking a complete medical history” and “vegetative anamnesis.” The risk-related anamnesis was in the mid-range with no significant differences between the groups (see table 2 (Tab. 2)). In contrast, self-assessment of the ability to give structured feedback showed a lower increase in the SP-Lecturer group than in the SP-Tutor group, whereby all of the groups already demonstrated a high value for this category at the beginning of the course (each with a minimum of 4.5 of 6 points).

No significant differences in performance on the exam questions were seen between the SP-Lecturer group and SP-Tutor group or between the SP-Tutor group and the RP-Tutor group (*Mdn*=4, *p*>.359, *N*=79). The poorest performance was seen in the responses to the open question on the seven dimensions of a symptom: A third (32.9%) correctly listed the two missing dimensions; somewhat more than a quarter (26.6%) were unable to list any dimension.

OSCE performance does not correlate with the performance on the exam questions (*r*=.03, *p*=.783). Likewise, there was no correlation between OSCE performance and learning success or the self-assessed level of knowledge at the end of the course.

#### 3.6. Course evaluation

The mean grade assigned to the course by students was a 1.9 on the conventional German academic grading scale (*SD*=0.6), whereby no differences existed between the comparative groups (*p*>.53). A large majority (87.8%) completely agreed with the statement that “Communication is of central importance to the medical profession.” Only a quarter of respondents completely agreed with the statement that “In general, communication can be learned.”

The majority of students (84.7%) completely agreed or mostly agreed that the communication course had been “useful and helpful.” Agreement was higher the more students reported having felt they benefited from feedback given by fellow students (*r*=.45) or by the instructor of the small group (*r*=.36, *p*<.001).

All of the groups displayed satisfaction with the leadership of the small group either by lecturers or tutors (*Mdn*=6). Satisfaction with course leadership correlates significantly with the claim of having benefited from lecturer/tutor feedback (*r*=.460, *p*<.001). However, when analyzing the subgroups, this correlation held only for the small groups taught by a tutor (SP-Tutor: *r*=.61, *p*=.001; RP-Tutor: *r*= .59, *p*<.001), but not for the lecturer-taught group, *r*=.02, *p*=.919, whereby the correlations SP-Lecturer and SP-Tutor significantly differed, *z*=-2.38, *p*<.017.

A total of 85.7% of the students partially, mostly or completely agreed with the statement that they would have liked to have had more practice with an SP. In contrast, only 37.8% of the students agreed with the statement that they would have liked more role-playing with their fellow students. There is a significant correlation between the desire for more role-play with SP and the claim of having benefited from receiving feedback from the SP (*r*=.25, *p*=.008).

## 4. Discussion

Students who were trained mainly with SP achieved better results on the OSCE if the course had been taught by a lecturer. In contrast, no significant difference was found between the groups regarding the overall learning success or the exam questions.

As a consequence, this study belongs to the few that have indeed been able to detect a measurable difference in the effectiveness of teaching between lecturers and tutors [7]. Because student satisfaction in the lecturer-taught groups was connected somewhat more weakly with whether the students had benefited from instructor feedback, other aspects must be responsible for the better results: While students felt more comfortable interacting with tutors, the natural authority of the lecturers (age, professional experience) possibly made the students take the instruction more seriously [25]. Student questions are presumably answered more precisely and accurately by lecturers. Schmidt et al. showed that by cleverly answering a question with another question, lecturers motivate students to hold more discussions among themselves [13]. Presumably, students receive stronger guidance in this case and attain deeper knowledge and understanding of the topic. Tutors often answer difficult questions directly thereby depriving students of the opportunity to figure out the larger context by themselves [25]. It is conceivable that this difference, if it is relevant, can be minimized through better training of the tutors. Although not directly tested here, it is generally conceivable that clear differences exist between the results of the different tutors and the different lecturers, which in turn would suggest that the preparation, training and supervision, as well as the opportunities for improvement in these areas, should be studied more closely.

As postulated in the secondary hypothesis, the OSCE scores achieved by the students in the RP-Tutor group were significantly better than those achieved by the students in the SP-Tutor group. This finding aligns with direction of the results seen by Bosse et al. [22]. When analyzed more closely, the present study is able to show, above all, a difference regarding the psychosocial and risk-related anamnesis. Both groups rated their history-taking skills as being better after completing the course, with the increase in both groups being about the same.

The OSCE result for the tutor-led groups suggests that students benefited from the teaching of psychosocial and risk-related anamnesis using role-playing more than from using SP. An explanation for this could be that switching roles and/or personal experience fostered empathetic responses when handling these potentially sensitive topics (alcohol consumption, nicotine use, family situation). In fact, studies show that empathy can be encouraged through role-playing [26].

An alternative explanation is that role-playing in the tutor-led groups is at first the better choice and that SP should be deployed later on. Role-playing offers a safe environment in which to practice [26]. The same is true for peer-teaching [14]. It is possible that the interplay between these two factors is especially effective at the beginning of a module if students are still not yet feeling quite so confident.

Conducting interviews with SP was more popular than role-playing, a finding similar to that of Gilligan et al. [27], who also saw a desire for more role-playing expressed by only a small percentage of students.

The OSCE performance does not correlate with the performance on the exam questions. This conforms to the assumption that a test of knowledge measures a different academic aspect than an OSCE, which, for instance, assesses complex communication skills [28].

The limitations of this study arise from the different group compositions in the SP-Lecturer and SP-Tutor groups: because the SP-Lecturer group had a higher percentage of women, there was the possibility that the higher OSCE scores are due to the better communicative abilities of women [29]. However, overall in this sample there was no difference in the OSCE performance in terms of sex. Also, it was impossible to trace some of the codes because either they had been incorrectly generated by the students or they were missing. Another limitation is that when adjusting the alpha levels for all tests, the group differences would no longer be significant. When only adjusting for the tests carried out for the overall OSCE score using the Bonferroni-Holm procedure, significant group differences continued to be visible at least regarding the comparative groups (*p*=.034 and *p*=.041). In addition, no preceding analysis of variance was carried out to compare the three groups. The exclusively pairwise comparisons of the groups according to SP-Lecturer vs. SP-Tutor and SP-Tutor vs. RP-Tutor were chosen, since in the universal comparison of the SP-Lecturer and RP-Tutor groups two independent variables would have differed at the same time (number of role-plays and course units with SP and group leadership). Since the two factors were not fully permuted, no two-way ANOVA is possible. Nevertheless, when interpreting the data, it should be noted that presumably there would have been no significant OSCE differences between the three groups if ANOVA had been preferred over pairwise comparisons. Future study designs should include teaching by lecturers with increased used of role-playing.

## 5. Conclusion and outlook

This study shows that in history-taking courses students who trained an increased number of times with SP benefited more from instruction by a university lecturer and that groups led by peer tutors benefited more from the use of RP. Regardless of the teaching method, students subjectively perceived a clear gain in learning as a result of attending the history-taking course. In particular, the learning progress seen in taking a complete medical history – the core objective of the course – underscores the relevance of this topic in the curriculum. There could be multiple reasons why students who have been taught by lecturers score more points on the practical assessment. Future studies should focus on possible factors linked to lecturers that positively impact student performance, such as professional experience or the use of specific pedagogical techniques. Alongside conventional methods, like having university lecturers teach courses, the use of tutors and role-play – as they are already practiced in many places – make sense when resources are in short supply. Further studies should aim to clarify which methods are most effective at which points during a history-taking course, and whether a certain combination of lecturers and tutors with SP and RP can each be effective and conducive to meeting objectives in a longitudinal curriculum.

## Profiles

**Name of school:** University of Würzburg and Würzburg University Hospital (UKW)

**Study program/occupation: **Medicine

**Number of students per year and/or per semester: **140-150

**Has a longitudinal curriculum covering communication been implemented? **Yes

**At which semester levels are communicative and social competencies taught? Which teaching formats are used?**

- 2^nd^ semester (doctor-patient relationship, subject: medical psychology and sociology)
- 3^rd^ or 4^th^ semester (doctor’s visit in psychiatry, subject: elective on bedside teaching)
- 5^th^ semester (medical history-taking, subject: practical clinical examination techniques)
- 6^th^ semester (brief intervention for risky alcohol consumption/smoking, subject: compulsory subject prevention; suicidality, subject: practical clinical skills)
- 7^th^ semester (“breaking bad news“, subject: interdisciplinary knowledge and action)
- 9^th^ semester (communication with family members of palliative patients about changes in the aim of care, subject: compulsory subject palliative care)
- 10^th^ semester (patient perspectives on breast cancer, subject: gynecology)

**Which teaching formats are used?** Role-play conversations with simulated patients or fellow students or real patients, bedside teaching, small group work, peer teaching

**During which semesters are communicative and social competencies tested (formative, pass/fail, graded)?**

- 3^rd^ or 4^th^ semester oral exam
- 5^th^ semester (history-taking OSCE station, pass/fail, graded)
- 6^th^ semester written questions prevention (pass/fail, graded)
- 10^th^ semester practical year OSCE (starting SS 2020 or WS 2020/21)

**Which assessment formats are used?**

- oral exam
- OSCE
- written test

**Who (e.g. hospital, institution) is in charge of development and implementation?** Professors and teaching coordinators from various departments of the University of Würzburg and Würzburg University Hospital (medical psychology, epidemiology, pediatrics, general practice, psychiatry, gynecology, palliative care), Skillslab, Institute of Medical Teaching and Medical Education Research

## Current professional roles of the authors

- Prof. Dr. med. Anne Simmenroth holds a chair at the Institute for General Practice at the University Hospital of Würzburg and is a practicing general practitioner. Her research focuses on the teaching and testing of communicative competence in medical studies, communication with non-German-speaking patients, and examination and training didactics.
- Julia Herchenröther is a medical student in her 10th semester. This paper is the topic of her dissertation.
- Elena Tiedemann is a psychologist (M. Sc.) and research assistant at the Institute for General Practice. Her research focuses on teaching communicative competencies in education. In addition to assisting with doctoral theses, she is involved in teaching anamnesis and prevention courses (including smoker counseling).
- Prof. Dr. phil. Heiner Vogel heads the Department of Psychotherapy and Medical Psychology at the Center of Mental Health of the University Hospital and the University of Würzburg. Research in medical psychology, psychotherapy, health care research, rehabilitation, and social medicine.

## Competing interests

The authors declare that they have no competing interests. 

## Supplementary Material

Questionnaire

## Figures and Tables

**Table 1:**
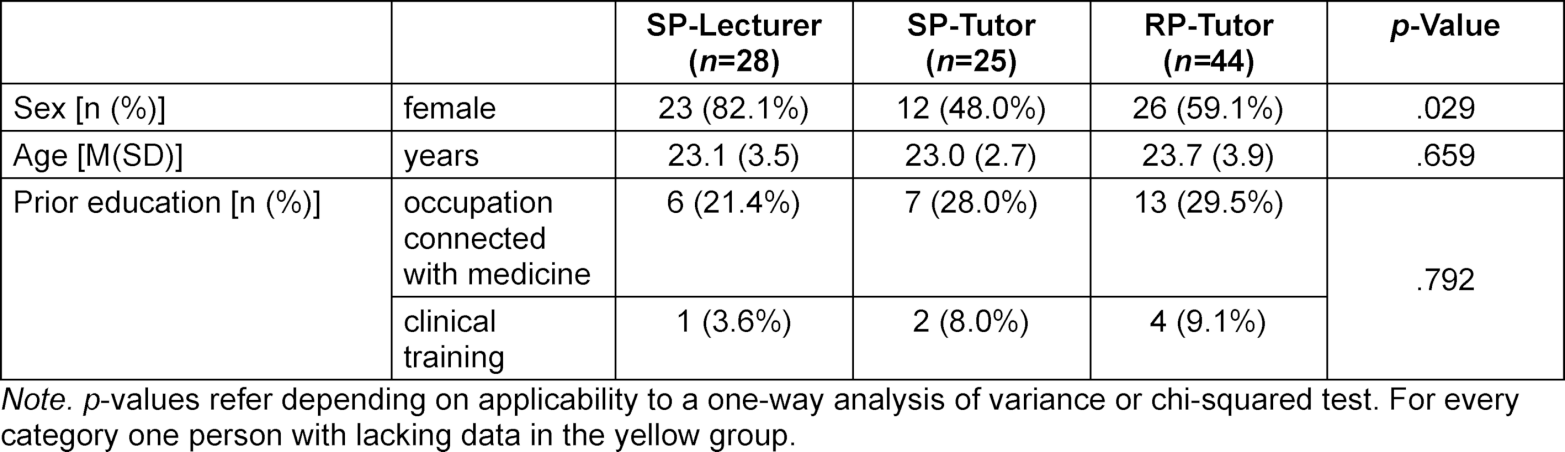
Demographic information of the three groups

**Table 2:**
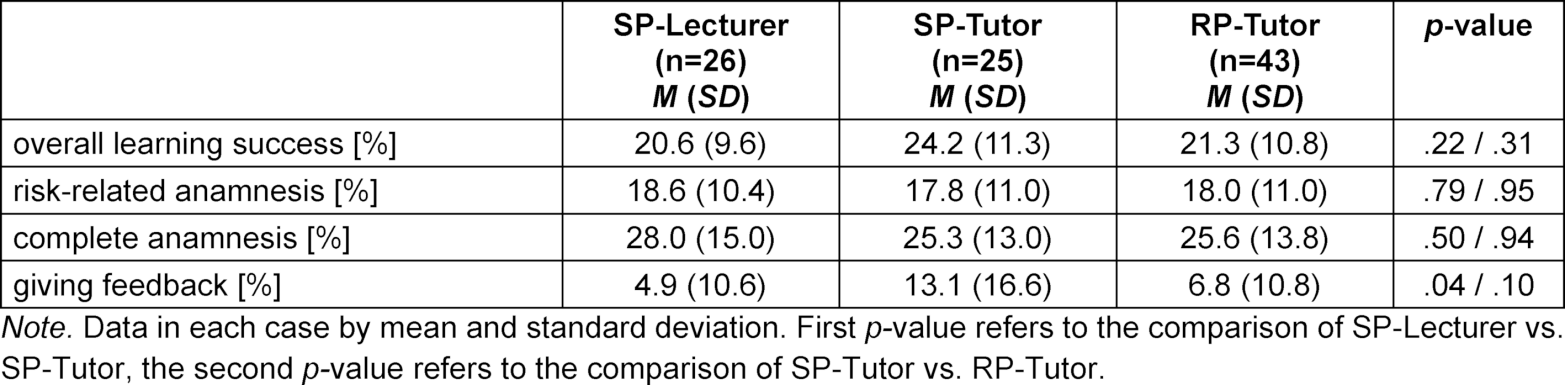
Gain in knowledge and skills of the three groups in the different categories
