# Supplementary material for: Which teaching method is more effective in a communication course – role-playing versus simulated patients, taught by tutors or faculty staff? A randomized trial
Source: GMS J Med Educ. 2021 Mar 15;38(3):Doc54. doi: 10.3205/zma001450 (PMC7994871; doi:10.3205/zma001450)
Supplement: Questionnaire [file JME-38-3-54-s-001.pdf]

MUSTER

EvaSys	Questionnare	Electric Paper

Institut für Allgemeinmedizin — Lang, Tiedemann, Simmenroth

PKU-Kurs Anamnese

Bitte so markieren: ☐ ☒ ☐ ☐ ☐ Bitte verwenden Sie einen Kugelschreiber oder nicht zu starken Filzstift. Dieser Fragebogen wird maschinell erfasst.
Korrektur: ☐ ■ ☐ ☒ ☐ Bitte beachten Sie im Interesse einer optimalen Datenerfassung die links gegebenen Hinweise beim Ausfüllen.

1. General information

1.1 Please create your **individual** code:

1. First and last letter of the first name of your **mother's grandmother** (e.g. "UA" bei UrsulA)
2. First and last letter of the first name of your **mother's grandfather** (e.g. "MN" bei MartiN)
3. Day of your **mother's** birthday (e.g. "03" at 3. october)

If you don't know the name of one of your grandparents please fill out "XX".

Please use block capital letters.

☐☐☐☐☐☐

1.2	Sex	☐ male	☐ female	☐ other
1.3	Age ☐☐			
1.4	Semester level ☐☐			
1.5	Prior education	☐ Occupation connected to medicine	☐ Clinical training	☐ None
1.6	Colour of my group was:	☐ red<br>☐ blue	☐ yellow	☐ green
1.7	My group was taught by:	☐ a tutor	☐ a lecturer	
1.8	During the second session my group trained with:	☐ simulated patients	☐ role-playing with fellow students	
1.9	How many history-taking interviews did you hold with simulated patients?	☐ 0<br>☐ 3	☐ 1	☐ 2
1.10	Did you swap a course?	☐ yes	☐ no	
1.11	Did you swap into a group of your own colour?	☐ yes	☐ no	

2. Communication and communication course

MUSTER

Attachment 1 to: Simmenroth A, Tiedemann E, Herchenröther J, Vogel H. Which teaching method is more effective in a communication course – role-playing versus simulated patients, taught by tutors or faculty staff? A randomized trial. GMS J Med Educ. 2021;38(3):Doc54. DOI: 10.3205/zma001450

2. Communication and communication course [Fortsetzung]

2.1	Communication is of central importance to the medical profession.	completely disagree ☐	☐	☐	☐	☐	☐ completely agree
2.2	In general, communication can be learned.	completely disagree ☐	☐	☐	☐	☐	☐ completely agree
2.3	The communication course was useful and helpful for me.	completely disagree ☐	☐	☐	☐	☐	☐ completely agree
2.4	I am satisfied with the instruction my group received from the lecturer/tutor.	completely disagree ☐	☐	☐	☐	☐	☐ completely agree
2.5	The theoretical part prepared me well for the role-plays.	completely disagree ☐	☐	☐	☐	☐	☐ completely agree
2.6	I would have wished for more role-plays with fellow **students**.	completely disagree ☐	☐	☐	☐	☐	☐ completely agree
2.7	I would have wished for more role-plays with **simulated patients**.	completely disagree ☐	☐	☐	☐	☐	☐ completely agree
2.8	The fellow students' feedback was of benefit for me.	completely disagree ☐	☐	☐	☐		☐ completely agree
2.9	The tutor's/lecturer's feedback was of benefit for me.	completely disagree ☐	☐	☐	☐		☐ completely agree
2.10	The simulated patient's feedback was of benefit for me.	completely disagree ☐	☐	☐	☐		☐ completely agree
2.11	The timeframe for the theoretical part was:	too tight ☐	☐	☐	☐	☐	☐ too extended
2.12	What grade would you give the course in total? (1-6)	1 ☐	☐	☐	☐	☐	☐ 6

2.13 Do you have any suggestions for improvement?

3. Personal communicative skills

To each of the statements we would like you to give **TWO** perceptions:

At first mark your **present** performance level (after completing the module) and then your performance level at the beginning of the course - that means you should assess your own performance level retrospectively **before** the beginning of the module.

Attachment 1 to: Simmenroth A, Tiedemann E, Herchenröther J, Vogel H. Which teaching method is more effective in a communication course – role-playing versus simulated patients, taught by tutors or faculty staff? A randomized trial. GMS J Med Educ. 2021;38(3):Doc54. DOI: 10.3205/zma001450

3. Personal communicative skills [Fortsetzung]

I can take a complete and structured medical history.

3.1	present self-assessment:	completely disagree	☐	☐	☐	☐	☐	☐ completely agree
3.2	retrospective self-assessment at the beginning of the course:	completely disagree	☐	☐	☐	☐	☐	☐ completely agree

I can take a complete vegetative anamnesis.

3.3	present self-assessment:	completely disagree	☐	☐	☐	☐	☐	☐ completely agree
3.4	retrospective self-assessment at the beginning of the course:	completely disagree	☐	☐	☐	☐	☐	☐ completely agree

I can take a complete medical history of current symptoms.

3.5	present self-assessment:	completely disagree	☐	☐	☐	☐	☐	☐ completely agree
3.6	retrospective self-assessment at the beginning of the course:	completely disagree	☐	☐	☐	☐	☐	☐ completely agree

I can take a complete psycho-social anamnesis.

3.7	present self-assessment:	completely disagree	☐	☐	☐	☐	☐	☐ completely agree
3.8	retrospective self-assessment at the beginning of the course:	completely disagree	☐	☐	☐	☐	☐	☐ completely agree

I can take a complete risk-related anamnesis.

3.9	present self-assessment:	completely disagree	☐	☐	☐	☐	☐ completely agree
3.10	retrospective self-assessment at the beginning of the course:	completely disagree	☐	☐	☐	☐	☐ completely agree

I can give structured feedback.

3.11	present self-assessment:	completely disagree	☐	☐	☐	☐	☐	☐ completely agree
3.12	retrospective self-assessment at the beginning of the course:	completely disagree	☐	☐	☐	☐	☐	☐ completely agree

I have mastered active listening.

3.13	present self-assessment:	completely disagree	☐	☐	☐	☐	☐	☐ completely agree
3.14	retrospective self-assessment at the beginning of the course:	completely disagree	☐	☐	☐	☐	☐	☐ completely agree

I am able to build a trusting doctor-patient-relationship.

3.15	present self-assessment:	completely disagree	☐	☐	☐	☐	☐	☐ completely agree

Attachment 1 to: Simmenroth A, Tiedemann E, Herchenröther J, Vogel H. Which teaching method is more effective in a communication course – role-playing versus simulated patients, taught by tutors or faculty staff? A randomized trial. GMS J Med Educ. 2021;38(3):Doc54. DOI: 10.3205/zma001450

MUSTER

3. Personal communicative skills [Fortsetzung]

3.16 retrospective self-assessment at the beginning of the course:	completely disagree	☐	☐	☐	☐	☐	☐ completely agree

I can start a doctor-patient-conversation appropriately (greeting, introduction, setting, etc.)

3.17 present self-assessment:	completely disagree	☐	☐	☐	☐	☐	☐ completely agree
3.18 retrospective self-assessment at the beginning of the course:	completely disagree	☐	☐	☐	☐	☐	☐ completely agree

I can recognize my patient's needs.

3.19 present self-assessment:	completely disagree	☐	☐	☐	☐	☐	☐ completely agree
3.20 retrospective self-assessment at the beginning of the course:	completely disagree	☐	☐	☐	☐	☐	☐ completely agree

I can respond to my patient's worries.

3.21 present self-assessment:	completely disagree	☐	☐	☐	☐	☐	☐ completely agree
3.22 retrospective self-assessment at the beginning of the course:	completely disagree	☐	☐	☐	☐	☐	☐ completely agree

I can summarize a doctor-patient-conversation at the end.

3.23 present self-assessment:	completely disagree	☐	☐	☐	☐	☐	☐ completely agree
3.24 retrospective self-assessment at the beginning of the course:	completely disagree	☐	☐	☐	☐	☐	☐ completely agree

Thank you for your help!

MUSTER

Attachment 1 to: Simmenroth A, Tiedemann E, Herchenröther J, Vogel H. Which teaching method is more effective in a communication course – role-playing versus simulated patients, taught by tutors or faculty staff? A randomized trial. GMS J Med Educ. 2021;38(3):Doc54. DOI: 10.3205/zma001450
